# The development of machine learning in bariatric surgery

**DOI:** 10.3389/fsurg.2023.1102711

**Published:** 2023-02-24

**Authors:** Bassey Enodien, Stephanie Taha-Mehlitz, Baraa Saad, Maya Nasser, Daniel M. Frey, Anas Taha

**Affiliations:** ^1^ Department of Surgery, GZO-Hospital, Wetzikon, Switzerland; ^2^ Clarunis, University Centre for Gastrointestinal and Liver Diseases, St. Clara Hospital and University Hospital, Basel, Switzerland; ^3^ School of Medicine, St George's University of London, London, United Kingdom; ^4^ Department of Biomedical Engineering, Faculty of Medicine, University of Basel, Allschwil, Switzerland

**Keywords:** machine learning, weight loss surgery, bariatric sugery, ML algorithms, systematic scoping review

## Abstract

**Background:**

Machine learning (ML), is an approach to data analysis that makes the process of analytical model building automatic. The significance of ML stems from its potential to evaluate big data and achieve quicker and more accurate outcomes. ML has recently witnessed increased adoption in the medical domain. Bariatric surgery, otherwise referred to as weight loss surgery, reflects the series of procedures performed on people demonstrating obesity. This systematic scoping review aims to explore the development of ML in bariatric surgery.

**Methods:**

The study used the Preferred Reporting Items for Systematic and Meta-analyses for Scoping Review (PRISMA-ScR). A comprehensive literature search was performed of several databases including PubMed, Cochrane, and IEEE, and search engines namely Google Scholar. Eligible studies included journals published from 2016 to the current date. The PRESS checklist was used to evaluate the consistency demonstrated during the process.

**Results:**

A total of seventeen articles qualified for inclusion in the study. Out of the included studies, sixteen concentrated on the role of ML algorithms in prediction, while one addressed ML's diagnostic capacity. Most articles (*n* = 15) were journal publications, whereas the rest (*n* = 2) were papers from conference proceedings. Most included reports were from the United States (*n* = 6). Most studies addressed neural networks, with convolutional neural networks as the most prevalent. Also, the data type used in most articles (*n* = 13) was derived from hospital databases, with very few articles (*n* = 4) collecting original data *via* observation.

**Conclusions:**

This study indicates that ML has numerous benefits in bariatric surgery, however its current application is limited. The evidence suggests that bariatric surgeons can benefit from ML algorithms since they will facilitate the prediction and evaluation of patient outcomes. Also, ML approaches to enhance work processes by making data categorization and analysis easier. However, further large multicenter studies are required to validate results internally and externally as well as explore and address limitations of ML application in bariatric surgery.

## Introduction

Machine learning (ML) is an approach to data analysis that makes the process of analytical model building automatic. The method is a category of artificial intelligence that relies on the ideology that systems can study information, recognize patterns, and derive decisions with little human intervention. The significance of ML stems from its potential to evaluate big data and achieve quicker and more accurate outcomes. ML has recently witnessed increased adoption in the medical domain. Rajkomar et al., claimed that ML has an advantage over traditional approaches since models learn from experience rather than prior programming (1). Moreover, a model developed by Sidey-Gibbons and Sidey-Gibbons showcased success in the medical domain of high accuracy, specificity, and sensitivity (2). Nonetheless, various shortcomings are associated with ML in the medical sector, including manipulation threats that could lead to misleading conclusions and privacy concerns that could cause leakage of patient data. A study by McCradden et al. highlighted ethical concerns as the primary limitation of ML algorithms, like discrepancies between patient trajectory and fair predictability (3). Despite the challenges, the potential of ML in the medical field is a growing field, and its success will mostly depend on comprehensive research and the development of solutions to the current limitations.

Bariatric surgery, otherwise referred to as weight loss surgery, reflects the series of procedures performed on people demonstrating obesity. A review of the benefits and threats of modern bariatric surgery revealed that it results in enhanced patient outcomes, especially for type 2 diabetics. Still, bariatric surgery triggers patient safety concerns and therefore, shared decision making and individual evaluation of advantages and disadvantages with patients are required (4).

Machine learning (ML) tools have grown in popularity among medical researchers over the past few decades. Some ML techniques have been demonstrated to produce quite precise forecasts and are being used more widely in the diagnosis and prognosis of various illnesses (5, 6). They have been frequently used to identify important aspects of patients' illnesses and model the course of the disease following therapy using complex medical data and health information (5–7).

Therefore, the main goal of this study is to explore the development of ML algorithms and their use in bariatric surgery. The review will cover studies that have examined the notion and their showcased results. It will highlight any advantages or shortcomings of ML use in bariatric surgery and suggest the future direction for researchers and surgeons.

## Material and methods

### Literature search and inclusion criteria

This review applied the suggestions of the PRISMA methodology for scoping studies (8). It contains 20 mandatory items and two voluntary variables the researcher conducting a scoping analysis must integrate into their manuscript. The eligibility criteria were: 1) Relevant articles published between 2016 and May 2022. Such papers were beneficial since they had a high chance of containing information pertinent to the research topic. 2) Articles published in English.

Choosing the appropriate files for the study required a comprehensive literature search. We searched various databases (PubMed, Cochrane, and IEEE) and search engines namely Google Scholar for articles published from 2016 to the current date. The literature search occured on May 8, 2022. Our team purified the search methods integrated through a group discussion. Furthermore, the authors scanned the references found in various articles to acquire additional documents for the study.

**Figure 1:**
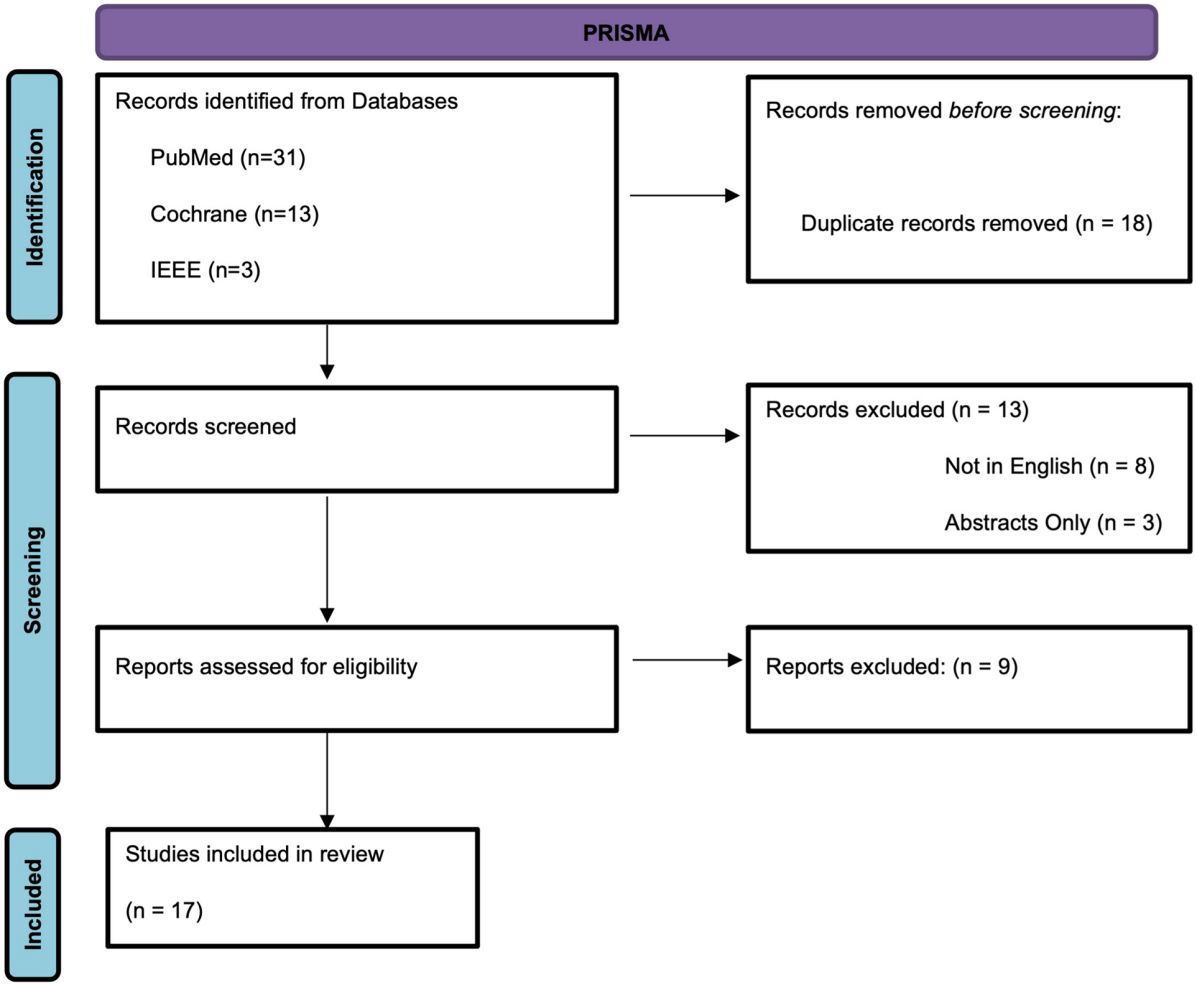
PRISMA diagram.

The search terms were “machine learning,” and “machine learning algorithms in bariatric surgery.” Also, we used phrases like “the development of machine learning in bariatric surgery,” “ML application in bariatric surgery,” and “ML in weight-loss surgery during the search process. On Pubmed, the applied filters were “best match” and “five years,” while on Cochrane and Google Scholar, the applied filters were “2016 to 2022”. All the researchers were involved in drafting the manuscript peer-reviewed the search plan by applying the PRESS checklist. This checklist facilitated the thorough evaluation of the consistency demonstrated during the process. The translation of the study questions ensured that the articles gathered were relevant to the study (see Figure 1).

### Data extraction

We generated a data logging tool highlighting the various features to consider when collecting data. The researchers examined contextual factors such as the type of surgery addressed by the articles. Also, we evaluated the topic of the publications to ensure that they were consistent with the research questions. The recency of articles guaranteed the collection of accurate information.

## Results

The search on the PubMed, Cochrane, IEEE databases and Google Scholar search engines identified 17 articles that qualified for inclusion in the study, as shown in Figure 1. The main content addressed by the documents was ML in bariatric surgery (see Table 1), whereby the researcher only included original studies discussing the matter. Out of the included studies, 16 concentrated on the role of ML in prediction, while only one addressed ML's diagnostic capacity. Most articles (*n* = 15) were journal publications, whereas the rest (*n* = 2) were papers from conference proceedings. Moreover, most included reports were from the United States (*n* = 6), as displayed in Table 1. Furthermore, most studies addressed neural networks, with convolutional neural networks as the most prevalent (see Table 2). Also, the data type used in most articles (*n* = 13) was derived from hospital databases, with very few articles (*n* = 4) collecting original data *via* observation.

**Table 1:** Characteristics and Summary of included articles.

Author	Year	Country	Primary Theme
Cao et al. (9)	2019	Sweden	Ensemble algorithms performed better than base approaches in predicting postoperative complications
Johnston et al. (10)	2019	USA	Patient-level prediction software was effective in assisting the patient selection to forecast successful type 2 diabetes treatment by obesity surgery
Assaf et al. (11)	2021	Israel	ML algorithms were effective in preoperative diagnosis of hiatal hernia
Chiong et al. (12)	2021	Australia	ML improved the prediction of body fat percentage among patients
Nudel et al. (13)	2021	USA	ML predicted gastrointestinal leakage and venous thromboembolism after surgery
Razzaghi et al. (14)	2019	USA	ML algorithms can predict bariatric surgery risks/outcomes even in imbalanced data sets
Thomas et al. (15)	2017	USA	Four neural networks showcased accurate predictions in long-term outcomes
Cao et al. (16)	2020	Sweden	Two out of three ML algorithms showcased limited success in predicting postoperative outcomes
Cao et al. (17)	2019	Sweden	Convolutional neural networks can successfully predict long-term health-related quality of life after surgery
Modaresnezhad et al. (18)	2019	USA	ML application in bariatric surgery helps in predicting surgical outcomes
Zhang et al. (19)	2020	China	ML can aid in predicting weight loss six months after surgery
Torquati et al. (20)	2022	USA	ML can help in reducing unnecessary readmissions
Cao et al. (21)	2020	Sweden	ML can predict long-term health-related life quality and comorbidities after surgery
Sheikhtaheri et al. (22)	2019	Iran	ML demonstrated success in predicting bariatric surgery patients’ complications
Weerakoon et al. (23)	2021	Sri Lanka	ML can help in the final and monthly predictions of patient weight after surgery
Dimeglio et al. (24)	2020	France	ML can help in categorizing weight loss and predicting potential weight gain after surgery
Sheidaei et al. (25)	2020	Iran	ML algorithm performed efficiently in predicting the various types of bariatric surgery based on the information patients present during their first physical exam

**Table 2:** Data types, sizes, and evaluation metrics of included articles.

Author	Data Type	Dataset Size (*n*)	Test Size (*n*)	ML Type	Evaluation Metrics
Cao et al. (9)	Derived	37,811	6,250	Eight bases and eleven ensemble algorithms	The accuracy and specificity of most algorithms were above 90%
Johnston et al. (10)	Observation	16,527	13,050	Patient-level prediction software	Internal discriminative accuracy and transportability of 95%
Assaf et al. (11)	Derived	2,482	2,482	3 CART Decision tree models	The models showcased a 92% specificity and a negative predictive value of 95%
Chiong et al. (12)	Derived	252	252	Improved relative error support vector machine (SVM)	Mean Absolute Error of 89%
Nudel et al. (13)	Derived	436,807	109,202	Gradient boosting machines (XGBs)	XGB was second-best in predicting leaks and venous thromboembolism
Razzaghi et al. (14)	Derived	Imbalanced data	Imbalanced data	Synthetic minority oversampling technique (SMOTE), random undersampling, and ensemble learning classification methods, including Random Forest, Bagging, and AdaBoost	SMOTE methodology is better than the random undersampling method
Thomas et al. (15)	Observation	478	144	Eight neural networks	Four neural networks showcased accurate predictions
Cao et al. (16)	Derived	37, 811	6,250	Multilayer perceptron (MLP), Convolutional neural network (CNN), and recurrent neural network (RNN).	MLP and CNN demonstrated a better performance than RNN
Cao et al. (17)	Derived	6,687	1,337	CNN	CNN showcased a better performance than the linear regression model
Modaresnezhad et al. (18)	Derived	120,000	120,000	Decision trees (DT), regression, and neural networks	Accuracy of 74%
Zhang et al. (19)	Observation	37	37	Siamese K-nearest neighbor	The framework showcased an accuracy of 83%
Torquati et al. (20)	Derived	393,839	393,839	Super learner algorithm	The algorithm demonstrated success in predicting readmissions
Cao et al. (21)	Derived	6,542	1,308	Bayesian networks	The algorithms showcased success
Sheikhtaheri et al. (22)	Derived	1,509	226	MLP network	Accuracy of 89%, specificity of 86%, and sensitivity of 91%within three months
Weerakoon et al. (23)	Observation	361	108	Artificial Neural Network (ANN)	Accuracy of 85% predicting post-operative final weight and 75% monthly weight
Dimeglio et al. (24)	Derived	795	381	Hierarchical cluster analysis (HCA)	The model showcased accurate predictions
Sheidaei et al. (25)	Derived	6,567	6,567	DT algorithm	Accuracy of 77% and sensitivity of 99%

### Summary of results

The study spearheaded by Cao et al. revealed that ensemble ML algorithms demonstrated a better performance than base algorithms in predicting postoperative complications among patients who have undergone bariatric surgery (9). Johnston et al. tested the predictive ability of ML in bariatric surgery forecasting successful type 2 diabetes treatment. The results indicated that the model was successful in predicting and assisting patient assortment (10). Assaf et al. argued that ML model applications improved pre-bariatric surgery diagnosis of hiatal hernia using contrast swallow studies and improved diagnostic sensitivity by 1.5 times baseline (11). Chiong et al. developed a support vector machine (SVM) model and that predicted the level of body fat percentage among patients with higher accuracy than other model compared (12). Nudel et al., developed an artificial neural network (ANN) capable of predicting postoperative gastrointestinal leakage better than traditional regression models (13). Razzaghi et al. posited that ensemble ML algorithms when applied with synthetic minority oversampling technique (SMOTE) have the greatest accuracy when predicting postoperative outcomes in bariatric surgery (14). Thomas et al. showed that four out of eight neural networks successfully predicted which patients had successful weight loss postoperatively after one year (15). Cao et al. argued that applying three unsupervised deep-learning neural networks led to improved but limited outcomes when predicting the occurrence of severe postoperative complications (16). The convolutional neural network algorithm also successfully predicted long-term health-related life quality after bariatric surgery (17).

Modaresnezhad et al., presented a semantic data integration, standardization and dimensionality reduction method that allowed for fast and efficient application of data mining techniques to large clinical datasets (18). Zhang et al. developed the Siamese-KNN ML model capable of predicting eventual weight loss six months after bariatric surgery, scoring close to 84% on accuracy (19). Torquati et al. posited that the Super learner ML algorithm outperformed traditional approaches like logistic regression in predicting thirty-day readmission risk after bariatric operations (20). Cao et al. revealed that Bayesian networks were appropriate tools for predicting long-term health-related life quality and comorbidities after bariatric surgery (21).

A different study spearheaded by Sheikhtaheri et al. employed a Clinical Decision Support system comprising MLP networks and predicted complications within ten days, one month, and three months after bariatric surgery with good accuracy and sensitivity (22). Weerakoon et al., argued that ML application in bariatric surgery could assist in weight prediction by tracking the pre-and post-surgery weight of patients with high accuracy (23). Moreover, Dimeglio et al. claimed that applying ML algorithms in bariatric surgery permitted the accurate categorization of individuals and predicted postoperative weight gain potential among patients (24). Sheidaei et al. indicated that the decision tree ML algorithm performed efficiently in predicting the various types of bariatric surgery based on the information patients present during their first physical exam (25).

## Discussion

The application of ML in bariatric surgery has gained much attention in previous years due to its capability to improve processes and ensure positive outcomes for patients. Most of the studies presented the primary application of ML to predict patient outcomes. Most research is based in 4 domains: Diabetes and BMI, postoperative complications, quality of life post-surgery and radiology.

### Diabetes and BMI

One of the best ways to achieve significant, persistent weight loss, improved glycemic control, and in many cases, remission of type 2 diabetes (DMII) is through bariatric surgery (26). Patients' ability to attain these results may be impacted by variables such as age, sex, medications, comorbidities, type of procedure, and prior weight-loss procedures (10). Johnston's Patient Prediction Model was able to allocate patients a probability of cessation of antihyperglycemic medications after bariatric surgery based on the preoperative factors above with high internal and external validity (10). To date, it has been difficult to use standard regression to predict long-term success in bariatric surgery patients (24). However, Weerakoon's model was able to predict the final weight of patients with 85% accuracy and monthly weight changes with 75% accuracy (23). Thomas et al. showed that using only pre-operative demographic, anthropometric, and comorbidity information, their neural networks determined which patients will have successful weight loss over a year postoperatively with 78% accuracy (15). These models can inform the clinicians on the selection of patients and allow clinicians to set more accurate expectations for the patients (10). Additionally, monthly weight prediction can allow for more consistent follow-up with patients, which is one of the main predictors of persistent weight loss (23, 27). DiMeglio's model was able to predict weight loss trajectories in a subset of patients with very high accuracy (24). If used during follow-up visits, this model can allow for the early identification of suboptimal weight trajectory and can allow for early and improved second-line physical, psychological, and nutritional management (24).

These models however are limited by their inability to predict multiple outcomes (10). This would mean the need for multiple models to provide a complete clinical application. An easily accessible application incorporating several of these ML models would be required (15). Another limiting factor is the difficulty encountered incorporating data from large databases due to logistical complexities and lack of standardization of heterogenous patient information (28, 29). To tackle these issues, Modersnezhad et al., developed the RxSem model, which is a system that integrates, standardizes, and mines data in medical databases by utilizing semantic networks for reducing data dimensionality and thus, making predictive analytics using large datasets feasible and efficient (18). Another limitation that must be addressed is how clinicians would incorporate the statistical predictivity of ML into their decision-making process. What would the threshold of predicted probability be at which a patient would be selected to undergo a procedure as opposed to below which they would not? Preference studies and benefit-risk analysis when paired with ML may provide a useful answer to this question (10). From an economic standpoint, combining ML and cost-benefit analysis can produce a “target efficiency” threshold at which limiting the intervention to those who meet the threshold will produce the greatest expected economic net benefit (10, 30, 31). A payer who wants to allocate limited resources while promoting economic efficiency could find this strategy helpful.

### Postoperative complications

Although bariatric surgery has lower mortality than other elective surgical procedures, its complications can be costly and severe (13, 32, 33). The stratification of postoperative complication risk can aid in patient selection, referral strategies, and patient counseling (13). It may also help detect high-risk patients for follow-up and management (13). Although traditional linear regression models can provide rather straightforward and understandable inferences, they have not yet been proven to be accurate and cannot thus be employed in clinical practice (9, 34, 35). Torquati et al., compared their “Super learner” algorithm outperformed traditional statistical models and demonstrated a higher AUC and sensitivity at predicting 30-day readmission risk postoperatively. With a large sample size of 393,833 patients, they showed that ML may be used to create tools that could aid clinicians to create targeted strategies that could minimize unnecessary readmission (20). Several other ML models were developed to predict postoperative outcomes, but which provided the most accurate results? Razzhagi et al., and Cao et al., were interested in answering this question. They tested several algorithms and ML models. They came to a similar conclusion that the ensemble algorithm when equipped with several classifiers and SMOTE provided the highest accuracy (9, 14). It is important to note that accuracy was high using this model but the sensitivity was low (9). Accuracy in and of itself is a function of incidence. If the incidence of postoperative complications is low, then accuracy will be high by default, and thus sensitivity is of more importance in rare outcomes (9). Further development and research are required before ML models can be applied to larger populations. Similarly, Nudel et al. developed an artificial neural network (ANN) that outperformed traditional regression models in predicting postoperative gastrointestinal leaks and similarly had high AUC and specificity, but its sensitivity was low (13). On the other hand, Sheikhtaheri et al., showed that their ANN when equipped with SMOTE can predict early complications of gastric bypass surgery with high sensitivity, and accuracy (22). Although their 89% sensitivity was high, it is important to note that they just considered total and not individual complications (22). This is due to low sample size and limited complications in their database (22). The rare nature of the complications and the issue of noncompliance with follow-up adds to the difficulty of creating informative models (36). It is important to note that from the studies above, only Cao and Torquati (9, 20) externally validated their results (9, 13, 14, 20, 22). This means that these models cannot yet be applied clinically until further research and external validation is demonstrated.

Cao et al., would later develop 2 deep level neural network models (DLNN) equipped with SMOTE capable of predicting outcomes with an AUC of 0.85 in the training dataset, however, it failed to predict them in the testing dataset with AUCs barely higher than a random guess at 0.57 (16). This would indicate that DLNNs are still far from being clinically applicable in everyday practice (16). The authors stated that the main benefit of DLNNs is they attempt to incrementally learn high-level characteristics from data (16). Hard-core feature extraction requires less human domain expertise, in contrast to classic ML techniques, and reduce the complexity of the data making patterns easier to see (16, 37, 38).

### Quality of life

The HRQoL is a broad, multifaceted term that encompasses important everyday functioning and subjective experience categories such as somatic sensation, physical functioning, social role functioning, and subjective well-being (39, 40). Cao et al. demonstrated that the DLNN “convolution neural network” (CNN) model showed an overwhelming advantage in predicting all the HRQoL measures when compared to multivariate linear regression models (MLR) in the context of postoperative bariatric surgery (17). These findings may be very helpful to patients' postoperative care and rehabilitation (17). Cao et al., later compared the use of Bayesian networks (BN) to their previous CNN model and found that the Gaussian BN outperformed both CNN and MRL in predictive accuracy (21). The authors assert that the BN model deserves future investigation in the future (21).

### Radiology

Machine learning models in radiological applications have been successfully used in the diagnosis and management of several medical fields related to the brain, breast, lung, and thyroid (41–44). In bariatric surgery, Assaf et al. utilized ML algorithms to increase the sensitivity of preoperative contrast swallow studies when evaluating patients for the presence of hiatal hernias (11). This ability can enhance conventional medical diagnosis and could reduce the number of patients needing hiatal exploration during bariatric surgery (11). Zhang et al., used functional magnetic resonance imaging (fMRI) with baseline whole-brain resting-state functional connectivity (RSFC) to develop a multivariate prediction framework “K-nearest neighbor (KNN)” (19). The Siamese-KNN achieved an accuracy of 83.78% and showed that neuroimaging biomarkers can be used to predict individual weight loss post-surgery and assist in personalized diagnosis for treatment of obesity (19). These applications are still new and require further investigation and large prospective research to confirm their findings.

### Research gaps

Multiple research gaps remain evident in this review. For instance, there is a scarcity of publications addressing the integration of ML algorithms in bariatric surgery. The potential cause for this situation is the newness of the concept. Moreover, there is a lack of articles addressing the challenges of ML integration in bariatric surgery. Hence, it becomes challenging to understand the various shortcomings that trigger the minimal incorporation of ML algorithms into bariatric surgeries. Handling the identified research gaps is necessary to ensure the availability of more supportive information.

### Potential developments in the field

The potential of ML algorithms to reduce surgical complications and improve patient care showcases that their development is inevitable and the benefits of ML in bariatric surgery will trigger its wide application and development. The improvement of ML integration in bariatric surgery mainly depends on the efforts of researchers to conduct more studies and highlight the algorithms that are most appropriate to apply in the sector. This would involve improving techniques that facilitate the extraction of more granular data from various medical records (13). An easily accessible application incorporating several of these ML models would also be required for everyday use (15). ML is still far from being clinically applicable (16), however, the future is certainly promising.

## Conclusion

This study indicates that ML has numerous benefits in bariatric surgery, however its current application is limited. The evidence suggests that bariatric surgeons can benefit from ML algorithms since they will facilitate the prediction and evaluation of patient outcomes. Also, ML approaches to enhance work processes by making data categorization and analysis easier. However, further large multicenter studies are required to validate results internally and externally as well as explore and address limitations of ML application in bariatric surgery.
