# Corrigendum: The development of machine learning in bariatric surgery

**DOI:** 10.3389/fsurg.2023.1186466

**Published:** 2023-04-04

**Authors:** Bassey Enodien, Stephanie Taha-Mehlitz, Baraa Saad, Maya Nasser, Daniel M. Frey, Anas Taha

**Affiliations:** ^1^ Department of Surgery, GZO-Hospital, Wetzikon, Switzerland; ^2^ Clarunis, University Centre for Gastrointestinal and Liver Diseases, St. Clara Hospital and University Hospital, Basel, Switzerland; ^3^ St. George’s University of London, London, United Kingdom; ^4^ Department of Biomedical Engineering, Faculty of Medicine, University of Basel, Allschwil, Switzerland

**Keywords:** machine learning, weight loss surgery, bariatric surgery, ML algorithms, systematic scoping review

A Corrigendum on The development of machine learning in bariatric surgery By Enodien B, Taha-Mehlitz S, Saad B, Nasser M, Frey DM and Taha A. (2023) Front. Surg. 10:1102711. doi: 10.3389/fsurg.2023.1102711

**Incorrect Affiliation**

In the published article, there was an error in affiliations associated with Dr. Anas Taha and Dr. Daniel M. Frey.

**The incorrect affiliations are:**

Bassey Enodien^1^, Stephanie Taha-Mehlitz^2^, Baraa Saad^3^, Maya Nasser^3^, Daniel M. Frey^4^, Anas Taha^2,4^

^1^Department of Surgery, GZO-Hospital, Wetzikon, Switzerland.

^2^Clarunis, University Centre for Gastrointestinal and Liver Diseases, St. Clara Hospital and University Hospital, Basel, Switzerland.

^3^School of Medicine, St. George’s University of London, London, United Kingdom.

^4^Department of Biomedical Engineering, Faculty of Medicine, University of Basel, Allschwil, Switzerland.

**The correct affiliations are:**

Bassey Enodien^1^, Stephanie Taha-Mehlitz^2^, Baraa Saad^3^, Maya Nasser^3^, Daniel M. Frey^1^, Anas Taha^1,4^

^1^Department of Surgery, GZO-Hospital, Wetzikon, Switzerland.

^2^Clarunis, University Centre for Gastrointestinal and Liver Diseases, St. Clara Hospital and University Hospital, Basel, Switzerland.

^3^School of Medicine, St. George’s University of London, London, United Kingdom.

^4^Department of Biomedical Engineering, Faculty of Medicine, University of Basel, Allschwil, Switzerland.

The authors apologize for this error and state that this does not change the scientific conclusions of the article in any way. The original article has been updated.

